# Australians are not Meeting the Recommended Intakes for Omega-3 Long Chain Polyunsaturated Fatty Acids: Results of an Analysis from the 2011–2012 National Nutrition and Physical Activity Survey

**DOI:** 10.3390/nu8030111

**Published:** 2016-02-24

**Authors:** Barbara J. Meyer

**Affiliations:** School of Medicine, University of Wollongong, Northfields Ave, Wollongong, NSW 2522, Australia; bmeyer@uow.edu.au; Tel.: +61-2-4221-3459

**Keywords:** *n*-3 LCPUFA, dietary intakes, Australian 2011–2012 national nutrition and physical activity survey, recommended *n*-3 LCPUFA intakes

## Abstract

Health benefits have been attributed to omega-3 long chain polyunsaturated fatty acids (*n*-3 LCPUFA). Therefore it is important to know if Australians are currently meeting the recommended intake for *n*-3 LCPUFA and if they have increased since the last National Nutrition Survey in 1995 (NNS 1995). Dietary intake data was obtained from the recent 2011–2012 National Nutrition and Physical Activity Survey (2011–2012 NNPAS). Linoleic acid (LA) intakes have decreased whilst alpha-linolenic acid (LNA) and *n*-3 LCPUFA intakes have increased primarily due to *n*-3 LCPUFA supplements. The median *n*-3 LCPUFA intakes are less than 50% of the mean *n*-3 LCPUFA intakes which highlights the highly-skewed *n*-3 LCPUFA intakes, which shows that there are some people consuming high amounts of *n*-3 LCPUFA, but the vast majority of the population are consuming much lower amounts. Only 20% of the population meets the recommended *n*-3 LCPUFA intakes and only 10% of women of childbearing age meet the recommended docosahexaenoic acid (DHA) intake. Fish and seafood is by far the richest source of *n*-3 LCPUFA including DHA.

## 1. Introduction

Dietary fatty acids consist of saturated, monounsaturated, and polyunsaturated fatty acids (PUFA), where the PUFA comprise of omega-6 (*n*-6) and omega-3 (*n*-3) PUFA. The major dietary *n*-6 PUFA are linoleic acid (LA) and arachidonic acid (AA), whilst the major dietary *n*-3 PUFA are alpha-linoleic acid (LNA), eicosapentaenoic acid (EPA), docosapentaenoic acid (DPA), and docosahexaenoic acid (DHA) [1]. The *n*-3 long-chain PUFA (*n*-3 LCPUFA) comprise of EPA, DPA, and DHA, and are also referred to as the marine sources of *n*-3 PUFA. This distinction between LNA and *n*-3 LCPUFA is necessary because the majority of health benefits have been attributed to the *n*-3 LCPUFA rather than to LNA.

There are numerous health benefits associated with *n*-3 LCPUFA [2,3,4,5,6,7]. The vast majority of health benefits attributed to *n*-3 LCPUFA is in cardiovascular disease. The GISSI prevenzione trial showed that supplementation of 0.85 g of EPA and DHA per day in men who had a previous myocardial infarction resulted in 20% reduction in total death, 30% reduction in cardiovascular death and 45% reduction in sudden death [5]. There is emerging evidence for the benefits of *n*-3 LCPUFA in mental health [8] with the biological plausibility explained in the review by Parletta *et al*. [9]. Given these health benefits various organisations, including government organisations, have come up with recommended *n*-3 LCPUFA intakes for optimal health.

In Australia, the National Health and Medical Research Council (NHMRC) has nutrient reference values (NRV) which include recommended intakes for macronutrients and micronutrients for various age and gender categories [10]. The NHMRC NRV includes adequate intakes (AI) which is defined as the median intakes of the population and is not a recommended intake [10]. The NHMRC NRV suggested dietary target (SDT) intakes are recommended intakes for the prevention of chronic disease [10]. The SDT for *n*-3 LCPUFA is 430 mg/day for female adults and 610 mg/day for male adults [10] and these recommendations are based on the 90th centile of intakes from the Australian National Nutrition Survey conducted in 1995 (NNS 1995) [11].

The International Society for the Study of Fatty Acids and Lipids (ISSFAL) recommends 500 mg *n*-3 LCPUFA per day for cardiovascular health [12]. ISSFAL also has a separate recommendation for pregnancy and lactating women and this recommendation is to consume at least 200 mg per day of DHA based on a position paper by Berthold *et al.* [13].

Given these recommended intakes for *n*-3 LCPUFA, it is important to know what Australians are currently consuming and if they are meeting these recommendations. Therefore, the overall aims are to describe the current PUFA intakes; to compare the *n*-3 LCPUFA intakes to recommended intakes and to compare current intakes to previous intakes. The specific aims are: (1) to report on the macronutrient intake, including *n*-3 LCPUFA, per age category as published by Howe *et al*. [11]; (2) to compare the median and mean *n*-3 LCPUFA intakes from the 2011–2012-NNPAS from food and supplements per age category; (3) to compare the *n*-3 LCPUFA intakes to recommended intakes; (4) to determine if women of childbearing age met the *n*-3 LCPUFA and DHA recommended intakes during pregnancy; (5) to compare the actual adult Australian food intake from five different food groups to the respective *n*-3 LCPUFA intakes; and (6) to compare the current 2011–2012 NNPAS PUFA intakes, including *n*-3 LCPUFA intakes, to previous PUFA intakes from the NNS 1995 [11].

## 2. Results

### 2.1. Numbers of Subjects from the 2011–2012 NNPAS and the NNS 1995 Surveys

Table 1 shows the number of people surveyed for each age and gender category from both surveys. Overall the numbers are slightly lower in the 2011–2012 NNPAS except for the 12–18 years and the 65+ years categories.

### 2.2. The 2011–2012 NNPAS PUFA, LA, LNA, and n-3 LCPUFA Intakes per Day

Table 2 shows the PUFA, LA, LNA and the n-3 LCPUFA intakes per day (mean ± SEM) per age category and gender. Generally the PUFA intakes are higher in males than females for all age categories, except the *n*-3 LCPUFA intakes are higher in females aged 65+ years than males of the same age category. The *n*-3 LCPUFA intakes range from 133 mg per day to 494 mg per day.

### 2.3. Comparison of the Median and Mean n-3 LCPUFA Intakes from the 2011–2012 NNPAS (Figure 1)

**Figure 1:**
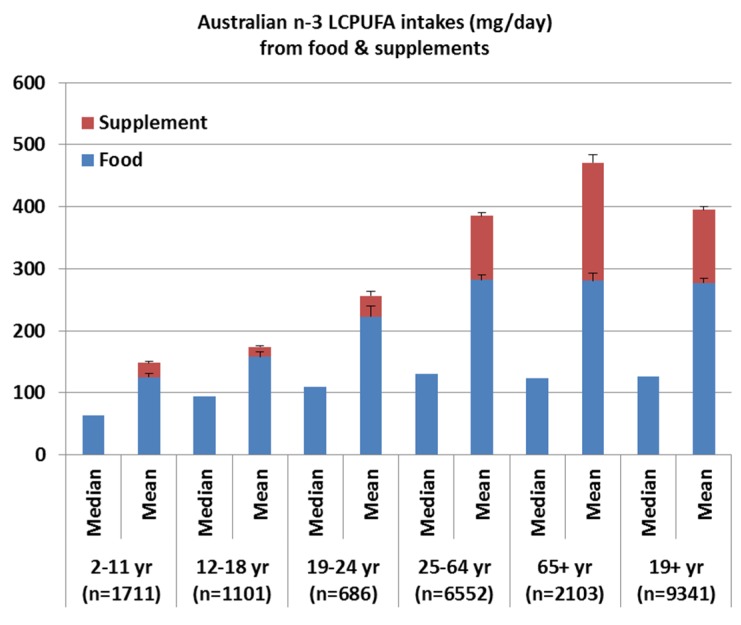
Comparison of the median and mean *n*-3 LCPUFA intakes from food and supplements per age category.

Figure 1 compares the median and mean *n*-3 LCPUFA intakes as well as the amounts coming from food and supplement sources. The median intakes are less than 50% of the mean intakes for all age categories, except for the 12–18 years olds which is 54% of the mean intakes. For adults (19+ years) the median intake is 32% of the mean intakes, which is largely driven by the 65+ years age category where the median intake is 26% of the mean intakes. This shows that *n*-3 LCPUFA intakes are highly skewed with few people consuming high amounts of *n*-3 LCPUFA and many people consuming low amounts of *n*-3 LCPUFA. The few people consuming large amounts of *n*-3 LCPUFA are consuming *n*-3 LCPUFA supplements. The proportion of *n*-3 LCPUFA coming from supplements is 27% for 25–64 years, 40% for 65+ years, and 30% of 19+ years and, therefore, the mean intakes are much higher than the mean intakes.

### 2.4. Comparison of n-3 LCPUFA Intakes to Recommended Intakes

#### 2.4.1. The Proportion of Adult Female and Male (19+ Years) Meeting the Recommended Intakes (Table 3)

The NHMRC NRV for *n*-3 LCPUFA SDT are based on the 90th centile of intakes from the previous National Nutrition Survey 1995 [11]. Less than a quarter of Australian adults are meeting the *n*-3 LCPUFA recommendations for optimal health. However, those adults consuming *n*-3 LCPUFA supplements approximately 50% are meeting the recommended intakes, whilst those not consuming supplements, only approximately 10% are meeting the recommended intakes.

When considering the median intakes, there was no contribution from *n*-3 LCPUFA supplements (Figure 1), as only 25% of adult women and 15% of adult men consumed *n*-3 LCPUFA supplements (Table 3).

#### 2.4.2. Adult Females of Childbearing Age (16–50 Years) *n*-3 LCPUFA and DHA Intakes across Centiles and Comparison to the ISSFAL Recommendations for DHA Intake (Figure 2)

**Figure 2:**
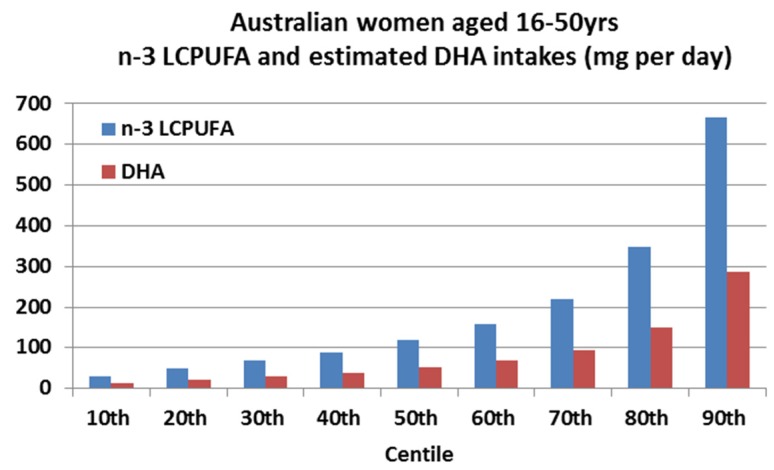
Australian women’s consumption of *n*-3 LCPUFA and the respective estimated docosahexaenoic acid (DHA) (mg per day) per centile.

There is a separate recommendation for pregnant women and the International Society for the Study of Fatty Acids and Lipids (ISSFAL) recommends at least 200 mg DHA per day during pregnancy and lactation [12,13]. Figure 2 shows the current consumption of *n*-3 LCPUFA and the respective estimated DHA intakes for women of childbearing age.

The median *n*-3 LCPUFA intake is 119 mg/day. DHA is estimated to be 43% of the total *n*-3 LCPUFA [11] and, therefore, the median DHA intake is estimated at 51 mg per day. Women in the 90th centile consume on average 287 mg DHA per day which meets the recommended DHA intake of at least 200 mg DHA per day.

### 2.5. Comparison of Adult Australian Intakes of food and the Respective n-3 LCPUFA in Those Foods

#### 2.5.1. Comparison of the Amount of Food Eaten (g per Day) by Adult Australians and the Respective Amount of *n*-3 LCPUFA Intakes (mg per Day) from the 2011–2012 NNPAS (Figure 3)

**Figure 3:**
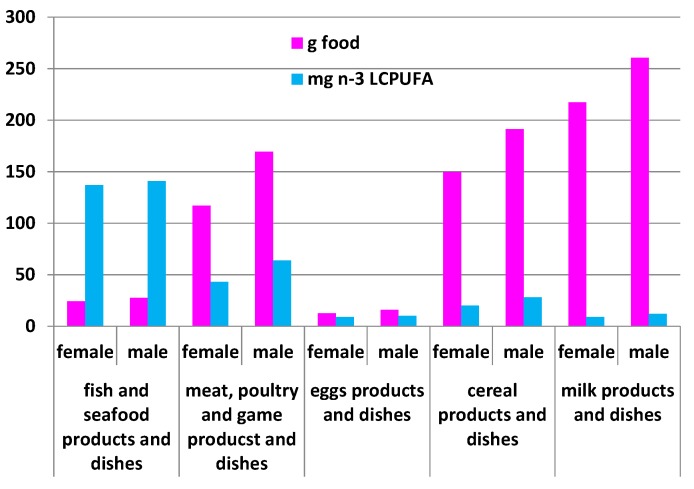
Comparison of the amount of food eaten (g per day) by adult Australians and the respective amount of *n*-3 LCPUFA intakes (mg per day) for five main food groups. Fish and seafood: fish, fish and chips, prawns, canned tuna, fish with pasta, paella with seafood; meat, poultry, and game: beef patty, steak, rabbit, offal, ham, lamb casserole, chicken stir-fry; egg products and dishes: eggs, omelette with cheese, spinach soufflé; cereal products and dishes: biscuits, cakes, pies (including meat pies), fried rice, pizza, *vol-au-vents*, quiche, gnocchi, lasagne, commercial hamburgers, croissants, pancakes; milk products and dishes: milk, yogurt, cream, cheese, ice cream, custard, milkshakes.

Figure 3 shows the amount of food eaten as grams per day by adult Australians and the respective amount of *n*-3 LCPUFA intakes as milligram per day. Female and male adults consume on average 24 g and 28 g of fish/seafood per day, respectively. Female and male adults consume on average 117 g and 170 g of meat, poultry, and game products and dishes, respectively, which are 4.5 and six times, respectively, higher than fish/seafood. Milk products and dishes and cereal products and dishes are consumed in greater quantities but make a small contribution to overall *n*-3 LCPUFA intakes. Eggs products and dishes are consumed the least in terms of gram amounts, but provide more *n*-3 LCPUFA per gram of food compared to milk products and dishes, and cereal products and dishes.

Approximately 16% of the Australian population consumes “nuts and nut products”. Seven percent of the Australian population consumes “peanuts and peanut products”, but peanuts do not contain *n*-3 fatty acids. Seven percent of the Australian population consumes “other nuts and nut product and dishes” and 2% consume “mixed nuts or nuts and seeds”, which would contribute to LNA, but not the *n*-3 LCPUFA.

#### 2.5.2. Comparison of the Adult Female and Male Mean Consumption *n*-3 LCPUFA Intakes (mg per g of Food) for the Various Food Groups (Figure 4)

**Figure 4:**
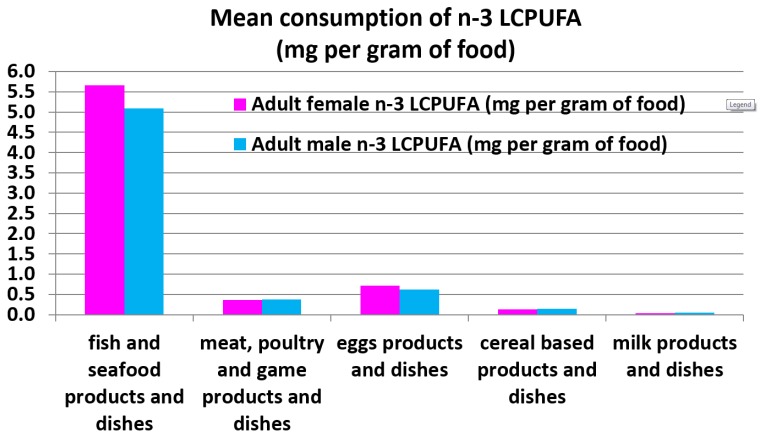
The actual adult female and males (19+ years) mean consumption of *n*-3 LCPUFA expressed as mg per gram of food for the various food groups.

Even though the consumption of fish and seafood is low (female and male mean intake of 24 g and 28 g, respectively), fish and seafood provide the largest amount of *n*-3 LCPUFA per gram of food (as shown in Figure 4).

When expressing the actual mean *n*-3 LCPUFA intakes per gram of food, fish and seafood products and dishes are 15-fold higher than meat, poultry, and game products and dishes; nine-fold higher than egg products and dishes; 38-fold higher than cereal-based products and dishes; and 114-fold higher than milk products and dishes.

### 2.6. Comparison of the 2011–2012 NNPAS and NNS 1995

#### 2.6.1. Comparison of the PUFA Intakes from the Two Australian National Nutrition Surveys: NNS 1995 and 2011–2012 NNPAS (Figure 5)

**Figure 5:**
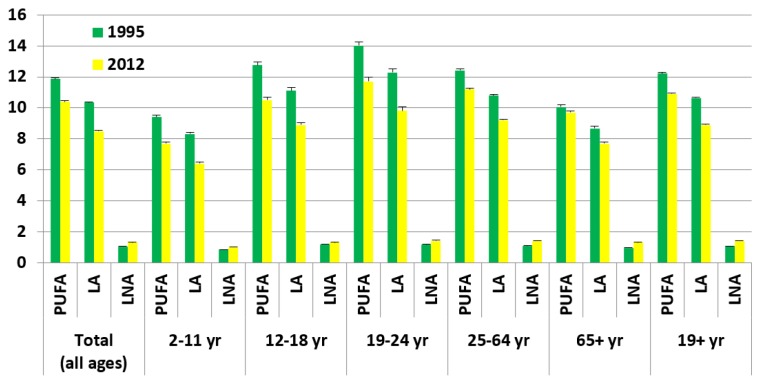
Comparison of the PUFA intakes (total PUFA, linoleic acid (LA), and alpha-linolenic acid (LNA)) (g per day) per age category.

As shown in Figure 5, the total PUFA and LA intakes have decreased from 1995 to 2012, but the LNA intakes have increased. For all ages, total PUFA decreased by 12%, LA decreased by 18% and LNA increased by 24%. These changes differed slightly between the different age groups but the general trend was the same.

#### 2.6.2. Comparison of the *n*-3 LCPUFA Intakes from the Two Australian National Nutrition Surveys: NNS 1995 and 2011–2012 NNPAS (Figure 6)

**Figure 6:**
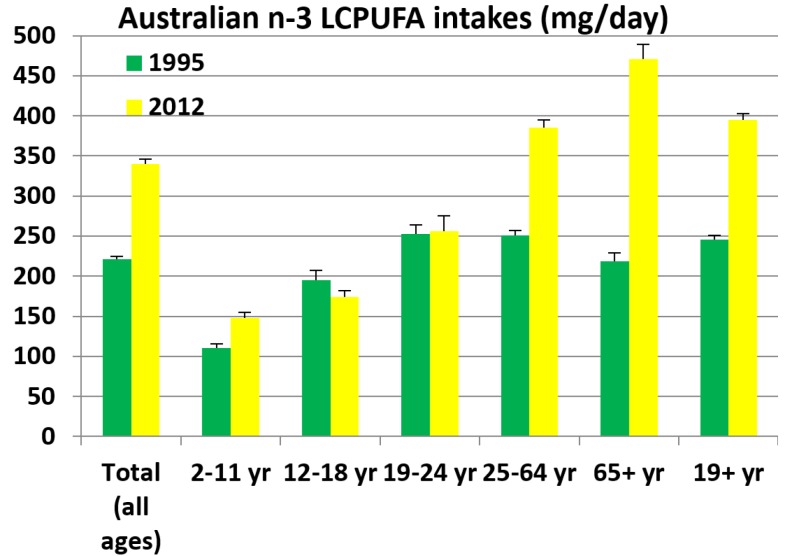
Comparison of the *n*-3 LCPUFA intakes per age category.

As shown in Figure 6, for all ages the *n*-3 LCPUFA intakes have increased by 54% from 1995 to 2012, with the greatest increase of 115% in the 65+ years age category. The younger adult age group (19–24 years) has not changed and there has been a slight 11% reduction in intakes in the 12–18 years category. The 2–11 years old age group has also increased their *n*-3 LCPUFA intakes by 35%.

## 3. Discussion

This study has shown that overall the Australian PUFA intakes have decreased since 1995, but there is an increase in omega-3 PUFA, LNA, and *n*-3 LCPUFA. These differences could be explained as follows. Different databases were used for the two surveys. The NNS 1995 used a custom food composition database which was developed by FSANZ (then known as ANZFA). The 2011–2012 NNPAS used an updated database from a custom food composition database prepared by FSANZ (AUSNUT 2011–2013). Differences in the nutrient values between the two databases reflect not only changes in the composition of foods, but also changes in available data and improvements in analytical methods over the period. Furthermore, a food model booklet was produced to aid in reporting of measures in 2011–2012 NNPAS. Food groups known to have been impacted by this change in methodology include “cereals and mixed dishes”, which could explain the differences in PUFA intakes in particular LA and LNA [14].

This increase, especially in *n*-3 LCPUFA is good as numerous health benefits have been associated with the consumption of *n*-3 LCPUFA [2,3,4,5,6,7]. Current mean *n*-3 LCPUFA intakes for adult Australians is 395 mg/day (277 mg/day from food and 118 mg/day from supplements), which have increased 1.6 fold since 1995, where the mean intakes were 246 mg/day. The increase can be explained by increased intakes in adults 25 years old or older, as there were no differences in *n*-3 LCPUFA intakes in the 19–24 years old category (Figure 6). The increase in *n*-3 LCPUFA can be probably explained by an increase in *n*-3 LCPUFA supplements, given that fish consumption (the major dietary sources of *n*-3 LCPUFA) has not changed from 26 g per day on average in NNS 1995 to 26 g in 2011–2012 NNPAS (24 g females and 28 g males). The NNS 1995 did not report on *n*-3 LCPUFA supplementation. However, currently the proportion of adults taking *n*-3 LCPUFA supplements is 25% for adult women and 15% for adult men (Table 3).

However, the current adult Australian *n*-3 LCPUFA median intakes of 126 mg/day (from food) and 154 mg/day (from food and supplements) are less than 50% of the mean intakes suggesting that the *n*-3 LCPUFA intakes are highly skewed and that there are some people consuming high amounts of *n*-3 LCPUFA, but the vast majority of the population are not consuming enough for optimal health. In fact when compared to recommended intakes only approximately 20% of the population met the recommendations for optimal health. A vast majority of adult Australians that were taking *n*-3 LCPUFA supplements met the recommended intakes (ranging from 46% to 67%); however, those people not taking *n*-3 LCPUFA supplements, only approximately 10% of the population met the recommended intakes. Given that approximately 20% of the population is consuming *n*-3 LCPUFA supplements, 80% of the Australian population is not meeting the recommended intake for *n*-3 LCPUFA for optimal health.

The current adult (19+ years) Australian *n*-3 LCPUFA intakes are higher than the US intakes of 113 mg *n*-3 LCPUFA intakes per day [15], but still falls far short of populations that consume high amounts of *n*-3 LCPUFA (810 mg per day, [16] and 905 mg per day [17]), such as the Japanese [16,17]. Fish and seafood is the richest source of *n*-3 LCPUFA and populations that consume fish/seafood, more so than meat and poultry, have higher intakes of *n*-3 LCPUFA. In the current Australian population the mean fish consumption is approximately six times lower than meat; yet consuming only one-sixth of the meat consumption from fish/seafood provides much more *n*-3 LCPUFA than consuming meat as shown in Figure 5. Moreover when the data is expressed as the mean consumption of *n*-3 LCPUFA as mg per gram of food (Figure 6), *n*-3 LCPUFA intakes are 15-fold higher than meat.

Fayet *et al*. [18] has conducted dietary modelling to achieve the Australian dietary recommended intakes for *n*-3 LCPUFA for all life stages [18] and the easiest way to achieve the recommended intakes is to consume fish and seafood. The most cost effective way of meeting these recommendations is to consume fish and seafood [18]. Fish may be expensive per kilogram; e.g., Atlantic salmon cost approximately $29 AUD per kilogram but this translates to 16 cents per 100 mg of *n*-3 LCPUFA, whilst meat costs $2 AUD per 100 mg *n*-3 LCPUFA (assuming meat costs approximately $25 AUD per kilogram) [18].

There appears to be a controversy about the efficacy of recent randomised controlled trials that assessed the health benefits of *n*-3 LCPUFA but there is an explanation for this. Randomised controlled trials and meta-analysis of trials up to the year 2002 demonstrated that supplementation with *n*-3 LCPUFA resulted in up to a 45% reduction in overall mortality from cardiac death [5,19]. However, reviews and meta-analyses of recent trials reported a lack of efficacy, suggesting that the *n*-3 LCPUFA are dead in the water [20,21]. Critical analyses of these recent trials have suggested that the lack of efficacy may be due to methodological problems [22]. Following the earlier successful trials, the American Heart Association issued guidelines for people with heart disease, suggesting consumption of at least two fish meals per week [23] in addition to fish oil supplements [24]. Between 2000 and 2010, the importation of fish oils into the USA escalated more than 10-fold from 2000 to 22,000 metric tonnes [25]. However, trials conducted after 2002 failed to screen people for high fish and/or fish oil supplement intake resulting in great variability of *n*-3 LCPUFA status across trial conditions [24]. Where screening did occur, the upper 50% of the control subjects and the lower 50% of the fish oil intervention subjects overlapped [26], suggesting that the two groups were not separated enough to demonstrate an effect in the test arm (*n*-3). Thus, future trials need to attenuate the possible ceiling effects created by high baseline, by taking blood samples to determine compliance and correlations between increased *n*-3 LCPUFA status and response.

It is well recognized that consumption of *n*-3 LCPUFA, especially DHA, is important for neurological development [27,28]. Previous data has shown the importance of DHA during the latter stages of pregnancy when the brain accrues its tissue mass [29]. More recently, however, it has been shown that DHA is also vital at the stage of when the neural tube closes at day 28 of gestation [30]. Due to its importance, there are specific recommendations for DHA intake for pregnant and lactating women. The Society for the Study of Fatty Acids and Lipids (ISSFAL) recommends at least 200 mg of DHA per day during pregnancy and lactation [12,13]. The current median *n*-3 LCPUFA and estimated DHA intakes in women from childbearing age are 119 mg per day and 51mg per day, respectively (Figure 4). In order to meet the recommended 200 mg DHA intake per day, only 10% of women (*i.e*., the 90th centile, Figure 4) are consuming enough DHA. An Australian study of pregnant women (*n* = 94) consumed a median DHA intake of 75 mg/day [31], which still falls short of the recommended 200 mg/day [12,13].

The main food sources of *n*-3 LCPUFA is from the fish and seafood category. Previous research in Australia has shown that meat and eggs also contribute to *n*-3 LCPUFA intakes [1,32], with meat contributing nearly 50% of the *n*-3 LCPUFA intakes [11]. This large contribution of *n*-3 LCPUFA intakes is not because meat is a rich source of *n*-3 LCPUFA but Australians consume at least six times more meat than fish/seafood. Yet consumption of only 26 g of fish seafood provides more than double the *n*-3 LCPUFA than if one was to consume approximately 150 g of meat. To really illustrate that fish/seafood is by far the richest source of *n*-3 LCPUFA, when the data is expressed as *n*-3 LCPUFA consumption as mg per gram of food, fish/seafood is 15-fold higher than meat (Figure 6).

The strengths of this study are (1) the large dataset (*n* = 12,153 from all ages 2+ years) that accurately reflects population intakes and (2) the ability to compare to previously published National Nutrition Survey intakes from 1995 per age/gender category to determine the changes in PUFA intakes. The weakness of this study is the use of 24-h recall data as fish/seafood consumption is not usually a frequently (daily) consumed food. However, to maximise the accuracy of the data, an average of the two 24-h recall data was used for all nutrients presented here, including *n*-3 LCPUFA. Furthermore, the NNS 1995 used a food frequency questionnaire (*n* = 8321) in addition to the 24-h recall data, and found no differences in *n*-3 LCPUFA intakes from 24-h recall data and the food frequency questionnaire [11]. Whilst the current 2011–2012 NNPAS did not use a food frequency questionnaire and hence the 24-h recall data cannot be checked against FFQ data, one could suggest that the use of 24-h recall data in a large population dataset assesses *n*-3 LCPUFA accurately, especially given that there was no difference in intakes between the 24-h recall and the FFQ in the NNS1995 [11].

## 4. Materials and Methods

### 4.1. The Australian Health Survey 2011-13 (AHS) Containing the 2011–2012 National Nutrition and Physical Activity Survey (2011–2012 NNPAS) Data from the Australian Bureau of Statistics (ABS)

In Australia, the Australian Health Survey 2011–2013 (AHS) was conducted by the Australian Bureau of Statistics (ABS) and they have released several publications on their website [32]. The AHS contains the 2011–2012 NNPAS and data was collected in 2011 and 2012 and reports made available on the ABS website [33].

Participants (*n* = 12,153 individuals aged two years and older) in the 2011–2012 NNPAS were interviewed by telephone and 24-h dietary recall of all food, beverages and supplements were recorded. Approximately eight days later, a second 24-h dietary recall was conducted by telephone interview. The interviewers used the automated multiple-pass method developed by the Agricultural Research Service of the United States Department of Agriculture [34]. For further detailed information on this dietary data collection, please refer to the ABS website [35].

### 4.2. NNS 1995 Survey Data

The NNS 1995 survey was conducted jointly by the Australian Bureau of Statistics and the then Department of Health and Family Services, with representation from rural and urban areas of all Australian states and territories [36]. Food intakes were surveyed in 13,858 individuals who were interviewed in their homes by qualified nutritionists using the 24-h dietary recall method; 8321 of them also completed a food frequency questionnaire.

### 4.3. Confidential Unit Record Files (CURF)

The CURF [37] contained unidentified information from each individual in the study, which included the individual’s identity number and demographic descriptors (including age and gender) as well as the amounts of each encoded food consumed by the individual in the 24-h recalls. Permission was sought to use the CURF data and hence the 24-h dietary recall data was obtained from the CURF and analysed as explained below.

### 4.4. Analysis of 2011–2012 NNPAS 24-h Recall Data

The mean (±SEM) of the energy and macronutrient intakes were tabulated by gender using the same age categories as the NNS1995 as reported by Howe *et al*. [11] for ease of comparison. The average of the two 24-h recalls was calculated for each individual and each nutrient. The mean (±SEM) intakes for all age groups and gender were calculated and are reported as total intakes and separated into food and supplement intakes. The median *n*-3 LCPUFA intakes for all age groups and gender were also calculated.

### 4.5. Comparison of n-3 LCPUFA to Recommended Intakes

The NHMRC NRV for the prevention of chronic disease has postulated SDT intakes for *n*-3 LCPUFA. The SDT are based on the 90th centile of *n*-3 LCPUFA intakes per gender; 430 mg/day for women and 610 mg/day for men [10]. The ISSFAL has recommendations for *n*-3 LCPUFA for cardiovascular health; ISSFAL recommends 500 mg/day [12].

From the 2011–2012 NNPAS, the proportion of female and male adults (19+ years) meeting the recommended *n*-3 LCPUFA was calculated for the total sample, plus those not taking and taking *n*-3 LCPUFA supplements.

From the 2011–2012 NNPAS, the women of child-bearing age (16–50 years) consumption of *n*-3 LCPUFA intakes were determined per centile from 10th to 90th centile. As DHA was not available from the 2011–2012 NNPAS, DHA was estimated to be 43% of the total *n*-3 LCPUFA [11] and, hence, also determined per centile from 10th to 90th centile. These data were compared to the recommended intake of at least 200 mg/day of DHA for pregnant and lactating women [12].

### 4.6. Comparison of Adult Australian Mean Food Intake and the Respective n-3 LCPUFA Intakes

The actual mean amount of food intake from the five main food sources of *n*-3 LCPUFA were compared to the respective mean amount of *n*-3 LCPUFA. The mean amount of *n*-3 LCPUFA (mg) was divided by the mean amount of food (g) to get *n*-3 LCPUFA expressed as mg per gram of food.

### 4.7. Comparisons of NNS 1995 and 2011–2012 NNPAS

The current PUFA intakes (2011–2012 NNPAS) were compared to the NNS 1995 PUFA intakes which were published previously by Howe *et al.* [11].

## 5. Conclusions

Adult Australian PUFA intakes (LA intakes) may have decreased, whilst *n*-3 PUFA intakes may have increased since 1995. However approximately 80% of Australians are not meeting the *n*-3 LCPUFA recommended intakes for optimal health and 90% of childbearing women are not meeting the recommendations for DHA intakes during pregnancy and lactation.

## Figures and Tables

**Table 1:** Total number of study participants per sex and age and category.

Age Category	2011–2012 NNPAS sample			NNS 1995 Sample		
	Female	Male	Total	Female	Male	Total
All ages	6451	5702	12,153	7242	6616	13,858
2–11 years	857	854	1711	950	971	1921
12–18 years	535	566	1101	522	564	1086
19–24 years	360	326	686	575	485	1060
25–64 years	3506	3046	6552	4137	3694	7831
≥65 years	1193	910	2103	1058	902	1960
≥19 years	5059	4282	9341	5770	5081	10,851

2011–2012 NNPAS: the 2011–2012 National Nutrition and Physical Activity Survey; NNS 1995: the National Nutrition Survey in 1995.

**Table 2:** 2011–2012 NNPAS polyunsaturated fatty acids (PUFA), linoleic acid (LA), whilst alpha-linolenic acid (LNA) and the *n*-3 long-chain PUFA (*n*-3 LCPUFA) intakes per day (mean ± SEM).

Age Category and Gender	PUFA (g)	LA (g)	LNA (g)	*n*-3 LCPUFA (mg)
Total F	9.5 ± 0.1	7.8 ± 0.06	1.2 ± 0.01	335 ± 9
Total M	11.4 ± 0.1	9.4 ± 0.08	1.4 ± 0.01	346 ± 9
2–11 F	7.1 ± 0.1	5.9 ± 0.12	0.9 ± 0.02	138 ± 10
2–11 M	8.2 ± 0.2	6.8 ± 0.13	1.0 ± 0.02	158 ± 11
12–18 F	9.6 ± 0.2	8.1 ± 0.22	1.2 ± 0.03	133 ± 7
12–18 M	11.5 ± 0.3	9.6 ± 0.22	1.4 ± 0.03	213 ± 15
19–24 F	10.1 ± 0.3	8.5 ± 0.28	1.2 ± 0.04	175 ± 14
19–24 M	13.4 ± 0.5	11.2 ± 0.42	1.6 ± 0.06	346 ± 36
25–64 F	10.2 ± 0.1	8.3 ± 0.09	1.3 ± 0.02	378 ± 14
25–64 M	12.4 ± 0.1	10.2 ± 0.12	1.5 ± 0.02	395 ± 14
≥65 F	9.1 ± 0.2	7.2 ± 0.13	1.2 ± 0.03	494 ± 26
≥65 M	10.5 ± 0.2	8.4 ± 0.17	1.4 ± 0.03	441 ± 24
≥19 F	9.9 ± 0.1	8.0 ± 0.07	1.2 ± 0.01	390 ± 11
≥19 M	12.1 ± 0.1	9.9 ± 0.10	1.5 ± 0.02	401 ± 12

F—Female; M—Male.

**Table 3:** Meeting the recommended intakes for *n*-3 LCPUFA with and without supplements.

Recommended Intakes for Females 19+ Years	Females 19+ Years (*n* = 5059)	No Supplements (*n* = 4054, 75%)	With Supplements (*n* = 1005, 25%)
>430 mg per day *	*n* = 1126 (22%)	*n* = 446 (11%)	*n* = 673 (67%)
>500 mg per day #	*n* = 1001 (20%)	*n* = 386 (9.5%)	*n* = 613 (61%)
**Recommended Intakes for Males 19+ Years**	**Males 19+ Years (*n* = 4282)**	**No supplements (*n* = 3625, 85%)**	**With supplements (*n* = 657, 15%)**
>500 mg per day #	*n* = 844 (20%)	*n* = 399 (11%)	*n* = 368 (56%)
>610 mg per day *	*n* = 702 (16%)	*n* = 326 (9%)	*n* = 302 (46%)

* National Health & Medical Research Council (NHMRC) nutrient reference values (NRV) suggested dietary target (SDT) intakes for adult females and males; # ISSFAL recommendations for cardiovascular health.

## Abbreviations

The following abbreviations are used in this manuscript:

MDPI: Multidisciplinary Digital Publishing Institute
DOAJ: Directory of open access journals
TLA: Three letter acronym
LD: linear dichroism
